# Surgical Resection of Hepatic Cystic Echinococcosis Impaired by Preoperative Diagnosis

**DOI:** 10.1155/2013/271256

**Published:** 2013-12-18

**Authors:** Tomohiko Yasuda, Hiroshi Yoshida, Junji Ueda, Yasuhiro Mamada, Nobuhiko Taniai, Masato Yoshioka, Akira Matsushita, Youichi Kawano, Yoshiaki Mizuguchi, Tetsuya Shimizu, Hideyuki Takata, Eiji Uchida

**Affiliations:** Department of Gastrointestinal Hepato-Biliary-Pancreatic Surgery, Nippon Medical School, 1-1-5 Sendagi, Bunkyo-ku, Tokyo 113-8603, Japan

## Abstract

Cystic echinococcosis (CE) is a rare afferent infectious disease in Japan. This paper reports a case of a hepatic cyst being diagnosed after surgical resection. A 40-year-old Syrian male was admitted for evaluation of a hepatic cyst. Serum antibodies of echinococcosis were negative. Enhanced computed tomography of the abdomen revealed a large cystic lesion, 9 cm in diameter, in the left lateral sector of the liver, which had many honeycomb-like septa and calcified lesions. Magnetic resonance imaging of this lesion revealed high intensity in the T2 weighted image. We preoperatively diagnosed this lesion as cystadenocarcinoma or CE and performed a left hepatectomy. Pathological examination revealed the presence of protoscolices in the fluid of the cysts and led to a diagnosis of this lesion as CE. In conclusion, on seeing patients with huge hepatic cysts who come from an epidemic area, we should consider hepatic CE.

## 1. Introduction

Echinococcosis is one of the most familiar zoonosis and mainly infects the human liver. The larval stage of *Echinococcus granulosus*, *E. multilocularis*, *E. shiquicus*, *E. oligarthrus*, *E. felidis*, and *E. vogeli* is known as a hydatid or echinococcosis [1]. They are transmitted through pet dogs and cats, and grow slowly in the human liver (70% of cases) and lungs (10% of cases) [2]. *E. multilocularis* and *Echinococcus granulosus* are important species for hygiene reasons. The former is epidemic in the northern hemisphere, north of the 38th parallel, and causes alveolar echinococcosis. The latter is epidemic in sheep raising areas all over the world; however, it is not endemic, except for afferent infections, in Japan and causes cystic echinococcosis (CE). In general, the diagnosis is not difficult to make using blood studies of serum antibodies and modern imaging techniques such as ultrasound, computed tomography (CT) and magnetic resonance imaging (MRI). However, in some cases, it is difficult to distinguish it preoperatively from hepatic and biliary tumors such as liver abscesses and mucinous cystadenocarcinoma [3]. This paper reports a case of hepatic CE being diagnosed by pathological findings.

## 2. Case Presentation

**Figure 1:**
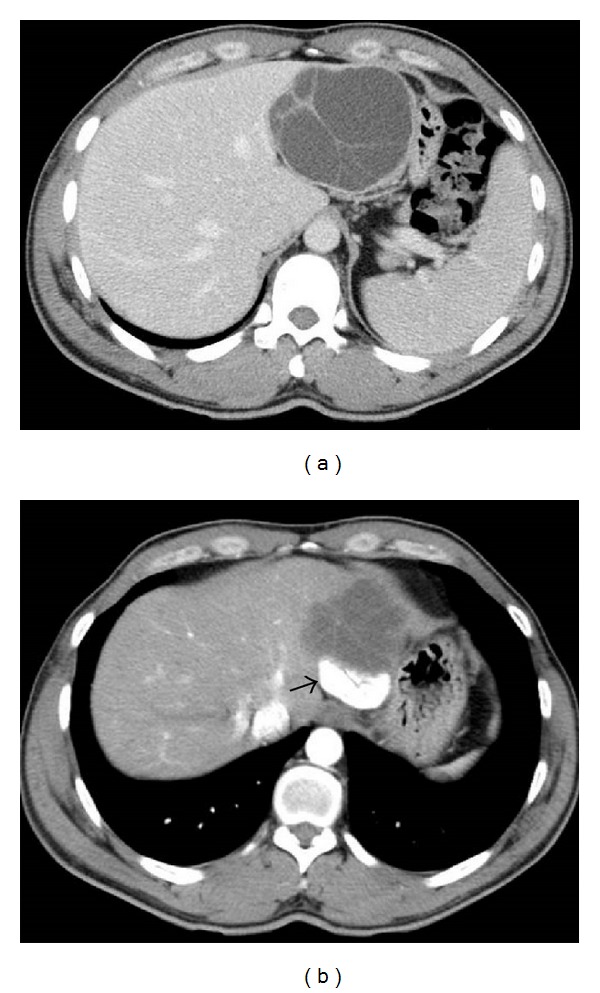
Enhanced computed tomography of the abdomen revealed a large cystic lesion, 9 cm in diameter, in the lateral segment of the liver, which has many honeycomb-like septa (a) with calcified lesions (b arrow).

**Figure 2:**
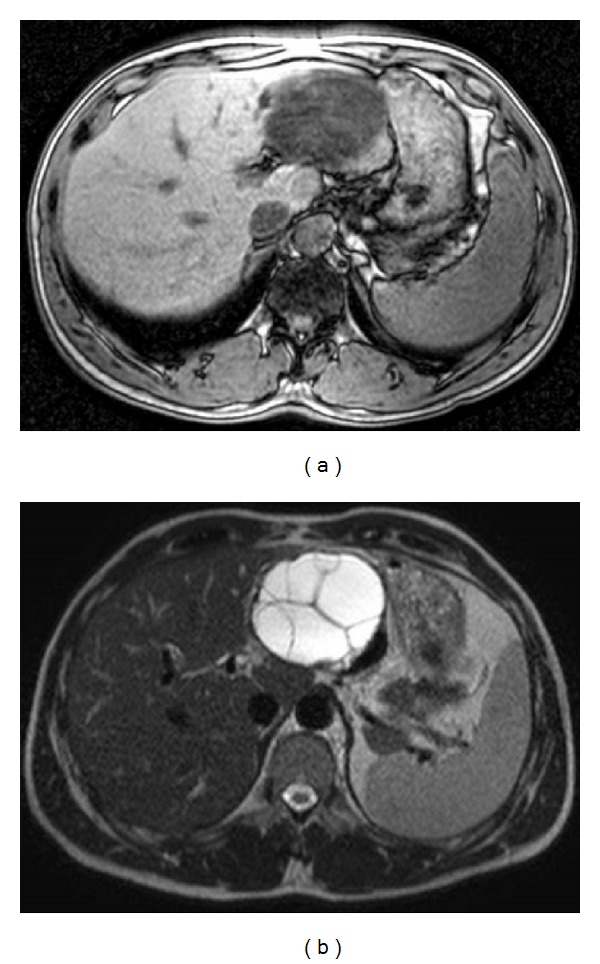
Magnetic resonance imaging of this tumor revealed low contrast intensity in the T1 weighted image (a) and high intensity in the T2 weighted image (b).

**Figure 3:**
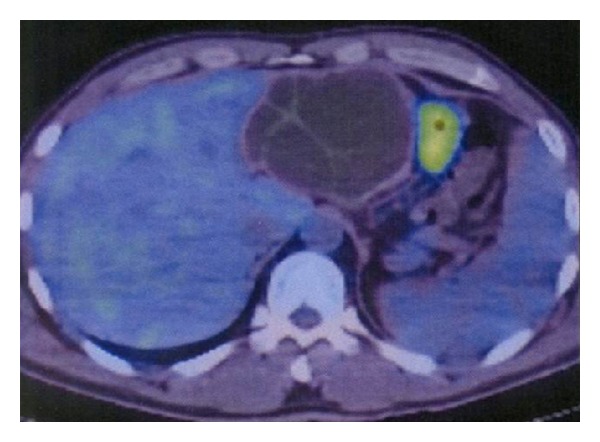
Positron emission tomography revealed no abnormal fluorodeoxyglucose uptake in the hepatic cyst or the cystic wall.

**Figure 4:**
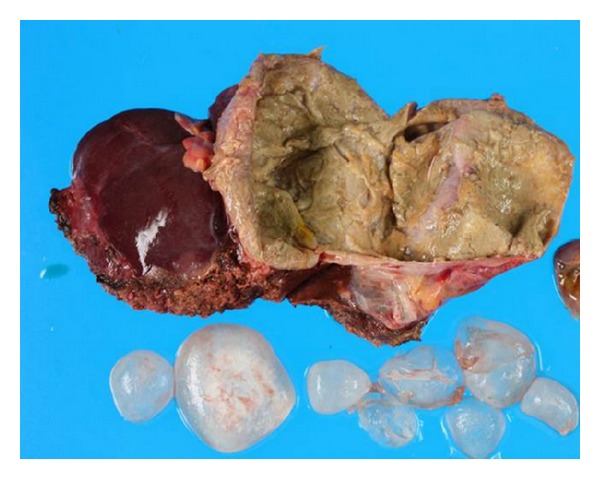
The resected specimen was a large cyst with a hard fibrous wall, which contained many small daughter cysts and watery fluid in their cavities.

**Figure 5:**
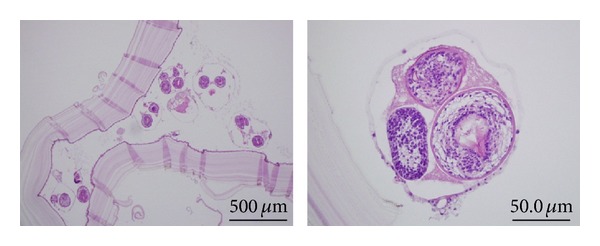
Microscopic examination revealed the presence of protoscolices in the fluid of the daughter cysts, which is characteristic of *Echinococcus granulosus*.

A 40-year-old Syrian male was admitted for evaluation of a liver cyst. The cyst was incidentally detected by a chest CT performed for a lung examination. The patient had no past history of any abdominal surgery and his vital signs were stable. Laboratory examinations showed a normal blood cell count and normal biochemical data: serum hemoglobin concentration of 16.3 g/dL (normal, 14 to 17 g/dL); platelet count of 14.0 × 10^4^/*μ*L (normal, 12 to 38 × 10^4^/*μ*L); total bilirubin level of 1.2 mg/dL (normal, 0.2 to 1.2 mg/dL); direct bilirubin level of 0.4 mg/dL (normal, <0.4 mg/dL); albumin level of 4.3 g/dL (normal, 3.8 to 5.5 g/dL); serum creatinine level of 0.81 mg/dL (normal, <1.2 mg/dL); and prothrombin time of 92.5% (normal, 70 to 130%), which indicated disease with a Child-Pugh score of Class A. The serum concentration of carcinoembryonic antigen was 1.8 ng/mL (normal, <2.5 ng/mL), and for CA19-9, it was 8.6 U/mL (normal, <37 U/mL). The serum antibodies of echinococcosis (enzyme-linked immunosorbent assay: ELISA) were negative. Enhanced CT of the abdomen revealed a large cystic lesion, 9 cm in diameter, in the left lateral sector of the liver, which had many honeycomb-like septa with calcified lesions (Figure 1). MRI of this lesion revealed low contrast intensity in the T1 weighted image and high intensity in the T2 weighted image (Figure 2). Positron emission tomography (PET) showed no abnormal fluorodeoxyglucose uptake in the hepatic cyst or the cystic wall (Figure 3). We diagnosed this cyst as either hepatic echinococcosis or mucinous cystadenocarcinoma. A left hepatectomy was attempted and the surface of the lesion in the lateral segment of the liver was hard. The other part of the liver was normal, and there was no ascites or dissemination in the abdominal cavity. After the left hepatectomy, fixation of the greater omentum to the peritoneum was performed to prevent delayed gastric emptying [4, 5]. The resected specimen had a large cyst with a hard fibrous wall, which contained many small daughter cysts with watery fluid in their cavities (Figure 4). Microscopic examination revealed the presence of protoscolices in the fluid of the daughter cysts, which is characteristic of *Echinococcus granulosus* (Figure 5). The cyst was diagnosed as CE. The postoperative course was uneventful. The patient was discharged on postoperative day 10. After 9 months, no recurrence was detected in enhanced CT of the abdomen and the patient was observed with no medication.

## 3. Discussion

Most hepatic cysts are asymptomatic, but complications, such as rupture [6], infection [7–9], biliary obstruction [10], and intracystic hemorrhage [11–15], can occur. The most frequent symptoms of hepatic CE are caused by compression due to the cyst swelling: abdominal pain (89.8%), abdomen lumps (10.9%), and cholangitis caused by cyst-biliary communication, such as pyrexia and icterus (5.5%) [16]. There are reports of anaphylactic reactions caused by a cyst rupturing. In this case, the patient did not complain of any symptoms at first presentation, but the cyst was detected by chance in a chest CT, which revealed long-standing abdominal postprandial swelling caused by the cyst.

The diagnosis consisted of imaging techniques and blood studies of serum antibodies. First, patients with hepatic cysts who come from an epidemic area are checked for the cysts characteristic of CE by CT or MRI imaging. Second, the diagnosis of CE is confirmed by the presence of serum antibodies for *Echinococcus *[3].

According to the World Health Organization Informal Working Groups on Echinococcosis (WHO-IWGE) classifications, CE is classified as CE1 (unilocular, anechoic cyst), CE2 (multiseptated, honeycomb cyst), CE3A (cyst with detached membranes), CE3B (cyst with daughter cysts in solid matrix), CE4 (cyst with heterogeneous content), or CE5 (solid cyst with calcifications) stages [2]. There are reports of cysts with many honeycomb-like septa, calcified lesions, membrane detachment, and many small inner cystic lesions with a lower density as characteristic findings [17]. In this case, the CT and MRI revealed a large cyst with calcified lesions, which had many honeycomb-like septa, and these findings corresponded to CE2. In addition, in Japan, the most frequently examined immunosorbent material is the crude antigen of *E. multilocularis*, which is extracted from a cyst of a cotton rat with alveolar echinococcosis. This is reacted with the patient's serum on a microplate after washout and is reacted with the antibody label alkaline phosphatase. This is colored after another washout, and the absorbance is measured (ELISA). Ito et al. reported that all alveolar echinococcosis cases gave positive reactions, whereas 2 of 32 cystic echinococcosis serum samples displayed weakly positive reactions in rEm18-ELISA. In this case, that is the reason why the serum antibodies (ELISA) were negative [18]. The material recommended by the WHO in serological diagnosis of CE is Antigen B. Furthermore, Ito et al. reported that the rapid immunochromatographic kit of Em18 and Antigen B, which is a protein refined from *E. multilocularis*, could make an accurate diagnosis of alveolar echinococcosis and CE17 [3]. On seeing patients with hepatic cysts who come from an epidemic area, if their imaging findings are compatible with CE and their serum antibodies of echinococcosis are negative, we should consider further examinations such as the rapid immunochromatographic kit.

The treatment of hepatic CE can be mainly surgical or percutaneous ultrasound-guided puncture, aspiration, injection, and reaspiration (PAIR) [2, 19]. The recommended therapy from the WHO guidelines is that stages CE1 (unilocular, anechoic cyst) and CE3A (cyst with detached membranes) without cyst-biliary communication are appropriate for PAIR, that stages CE2 (multiseptated, honeycomb cyst) and CE3B (cyst with daughter cysts in solid matrix) are feasible for PAIR and surgery, and that stages CE4 (cyst with heterogeneous content) and CE5 (solid cyst with calcifications) do not require treatment [2, 19]. PAIR is less invasive and enables a shorter hospital stay [2, 20]. However, complications develop in 25.2% of patients, including anaphylactic shock, secondary echinococcosis, and chemical cholangitis [20]. On the other hand, excisional surgeries such as cystpericystectomy and liver resection minimize the risk of recurrence, bile leak, and abscess [16]. In this case, we conducted a left lobectomy because we could not make a diagnosis of CE and considered that the cyst could be mucinous cystadenocarcinoma. When operating on an undiagnosed cyst, it is imperative not to spill the cyst fluid and to treat the cyst carefully.

## 4. Conclusion 

We encountered a case of CE with difficulties in making the preoperative diagnosis. We must always consider CE when treating patients with liver cysts who come from an epidemic area, conduct a Western blot examination if their serological testing (ELISA) is negative, and be prepared to perform a complete surgical resection without spilling the cyst fluid.
